# Sequential two-step chromatographic purification of infectious poliovirus using ceramic fluoroapatite and ceramic hydroxyapatite columns

**DOI:** 10.1371/journal.pone.0222199

**Published:** 2019-09-19

**Authors:** Yae Kurosawa, Shigehiro Sato, Tsuneo Okuyama, Masato Taoka

**Affiliations:** 1 R&D Department, HOYA Technosurgical Corporation, Akishima-shi, Tokyo, Japan; 2 Laboratory of Infectious Disease and Immunology, Department of Microbiology, Iwate Medical University, Shiwa, Iwate, Japan; 3 Protein Technos Institute, Atsugi-shi, Kanagawa, Japan; 4 Laboratory of Biophysics and Biochemistry, Department of Chemistry, Graduate School of Science and Engineering, Tokyo Metropolitan University, Hachioji-shi, Tokyo, Japan; Stanford University School of Medicine, UNITED STATES

## Abstract

Infectious virus purification techniques are important for vaccine development and gene therapy applications. However, the standardized one-step purification technique using ceramic hydroxyapatite (CHAp) has proven unsuitable for poliovirus. Therefore, we designed a sequential two-step chromatographic technique for purification of the infectious Sabin type 2 vaccine strain of poliovirus from the cell culture supernatant. In the first step, we removed protein contaminants from the Sabin type 2 virus fraction by pH gradient elution on a ceramic fluoroapatite column. In the second step, we removed double-stranded DNA derived from host cells by diluting the virus fraction, directly loading it on a CHAp column, and purifying it using a phosphate gradient with 1 M sodium chloride. This process achieved removal rates of more than 99.95% and 99.99% for proteins and double-stranded DNA, respectively, and was highly reproducible and scalable. Furthermore, it is likely that it will be applicable to other virus species.

## Introduction

The development of safer vaccines demands a reduction in their side effects; hence, regulatory authorities such as the Food and Drug Administration require both developers and manufacturers to improve the production and purification processes [1,2]. Various procedures have been developed for vaccine purification, including ultracentrifugation, liquid chromatography (LC), electrophoresis, precipitation, and molecular sieving. In particular, ultracentrifugation using cesium chloride, sucrose, or iodixanol gradients has been used to process several viral preparations for pharmaceutical use [3]. However, this procedure decreases the infectivity of some viruses [4] and is unable to separate particulate contaminants with physical characteristics similar to the virus particles, such as host cell DNA, from the virus fraction [5]. In addition, density-gradient separation is time-consuming and has low scalability and economic efficiency.

There is a current trend toward using LC for the purification of viral preparations because it is easy to use from the early capture stage to the final purification phase, and, unlike traditional centrifugal procedures, offers a straightforward scale-up. Furthermore, it has recently been used for large-scale purification (>10^13^ virus particles) using several approaches, including ion exchange, size exclusion, hydrophobic interaction, immobilized metal affinity, and hydroxyapatite chromatography [6–13].

For biosafety reasons, hydroxyapatite is commonly used in biomaterial engineering and regenerative medicine [14] as well as for the purification of pharmaceutical products [15]. Recently, our team standardized the hydroxyapatite chromatography procedure and successfully purified dengue virus type 2 and Japanese encephalitis virus from the cell culture supernatants of virus-infected C6/36 cells and mouse brain homogenate, respectively, using one-step hydroxyapatite LC [16,17]. Scanning electron microscopy analysis confirmed that the virus particles were bound to and released from the surface of the hydroxyapatite via the chromatographic processes, clearly indicating the phosphate-dependent absorption/desorption mechanism of this material [18,19]. Therefore, we considered that apatite-based materials would be suitable for the purification of vaccines for diseases and vectors for gene therapies, increasing their effectiveness and reducing their cost.

Two kinds of polio vaccines are currently available: the Salk and Sabin vaccines. The Salk vaccine is an injectable vaccine of formalin-inactivated virulent poliovirus strains, whereas the Sabin vaccine is an oral vaccine that uses live attenuated virus strains. The Sabin oral vaccine causes occasional incidences of vaccine-associated paralytic poliomyelitis by a circulating vaccine-derived poliovirus [20]; hence, the development of a noninfectious method of vaccination has become a priority. Consequently, an injectable Sabin vaccine has recently been developed from safer strains [21,22], and it is now possible for small companies to manufacture this vaccine with a smaller investment in facilities. This is because the Sabin strain is less transmissible than the other wild-type strains and does not require a high level of biosafety [23]. Polioviruses belong to acid-stable Picornaviridae and retain their infectivity at pH 3 and lower [24]. The spherical virus particles do not contain a lipid envelope, have a diameter of approximately 30 nm, and are composed of four structural proteins: VP1, VP2, VP3, and VP4. However, the standardized one-step virus purification procedure using ceramic hydroxyapatite (CHAp) is unsuitable for poliovirus. Therefore, here, we report on the design and experimental validation of a sequential ceramic fluoroapatite (CFAp)–CHAp LC procedure for purification of the Sabin type 2 strain of poliovirus.

## Materials and methods

### Preparation of Sabin type 2 virus-containing cell culture supernatant

Vero cells (the American Type Culture Collection, Manassas, VA, USA) were cultured in minimum essential medium (MEM; Thermo Fisher Scientific Inc., Waltham, MA, USA) containing 10% fetal bovine serum (FBS; Thermo Fisher Scientific Inc.) and L-glutamine (2 mM; Thermo Fisher Scientific Inc.) in 225-cm^2^ flasks (Sumitomo Bakelite Co., Ltd., Tokyo, Japan) at 37°C in 5% CO_2_ for 3 days. The medium was then changed to MEM containing 2% FBS and 2 mM L-glutamine, and the cells were grown for a further day. After changing the medium to MEM (63 mL) without FBS, Sabin type 2 virus was inoculated onto the cell monolayer at a multiplicity of infection of 0.01 and cultured at 37°C for 2 days. The cell culture supernatant was then collected, passed through a 0.45-μm filter (polyethersulfone membrane; Thermo Fisher Scientific Inc.) using vacuum to remove cell debris, and stored without any stabilizer at −80°C until use. The virus was highly stable under this condition and showed no change in its TCID_50_ value during the 4-month storage period (data not shown).

### CHAp and CFAp columns

CHT^™^ Ceramic Hydroxyapatite, Type II (CHAp; 40-μm particle size) and CFT^™^ Ceramic Fluoroapatite, Type II (CFAp; 40-μm particle size) were purchased from Bio-Rad Laboratories Inc. (Hercules, CA, USA). Both are ceramic-type materials with strict specifications and their pore sizes are 80–100 nm and 60–80 nm, respectively [25,26]. The particles were packed into empty stainless steel columns (4.6 mm i.d. × 35 mm; Sugiyama Shoji Co., Ltd., Kanagawa, Japan) in-house using a dry method.

### Chromatographic procedures

Chromatography was performed using a BioLogic DuoFlow^™^ system (Bio-Rad Laboratories Inc.) with a 10- or 20-mL sample loop at a flow rate of 1.0 mL/min. The samples were loaded onto a CHAp/CFAp column and eluted using a linear gradient of sodium phosphate buffer (NaPB) ranging from 10 mM to 600 mM, details of which are provided in the legend of each figure. The resulting eluate was monitored for ultraviolet (UV) absorbance at 260 and 280 nm and for conductivity. The collected fractions were kept at 4°C and were immediately used for the evaluations described below.

Before use, the running buffers were filtered (0.22 μm). The fraction collector was placed in a biological safety cabinet, and the attached tubes were autoclaved. In addition, the interiors of the pumps, columns, and lines were sterilized with approximately 80% ethanol (Amakasu Chemical Industries, Tokyo, Japan), washed with autoclaved ultrapure water followed by 600 mM NaPB, and equilibrated with 10 mM NaPB before use. After the experiments, the column and system were sterilized with 0.5 M NaOH for 10 column volumes and washed with autoclaved ultrapure water to remove the alkaline solution.

### Measurement of infectivity of the virus-containing fractions

The Sabin type 2 virus titer was obtained by measuring the median tissue culture infectious dose (TCID_50_) using a confluent monolayer of Vero cells in 96-well microplates. To prepare the confluent monolayer, Vero cells (100 μL, 1 × 10^5^ cells/mL) were cultured in MEM containing 10% FBS at 37°C for 1 day. Each fraction obtained by LC separation was 10-fold serially diluted with MEM containing 10% FBS, and the resulting diluents (50 μL) were inoculated into each well (*n* = 3) and cultured for 1 week. Each well of the microplates was then examined under a light microscope CKX31 (Olympus Corporation, Tokyo, Japan) to determine whether cytopathic effects had occurred. Titers were calculated using the Reed–Muench method [27].

### Additional procedures

The concentrations of double-stranded DNA (dsDNA) and proteins were determined using the Quant-iT^™^ PicoGreen dsDNA Assay Kit (Thermo Fisher Scientific Inc.) and the Micro BCA^™^ Protein Assay Kit (Thermo Fisher Scientific Inc.) according to the manufacturer’s instructions. For sodium dodecyl sulfate–polyacrylamide gel electrophoresis (SDS–PAGE), the LC fractions were concentrated using a centrifugal filter device (Ultracel YM-10; Millipore Corporation, Billerica, MA, USA) and applied to 15% polyacrylamide gel (c-PAGEL; ATTO Corporation, Tokyo, Japan). Each protein band was visualized by silver staining [28], and the molecular weight was calculated by the Gel Doc^™^ EZ Imager (Bio-Rad Laboratories Inc.).

## Results

### Two-step procedure for the purification of Sabin type 2 virus

**Fig 1:**
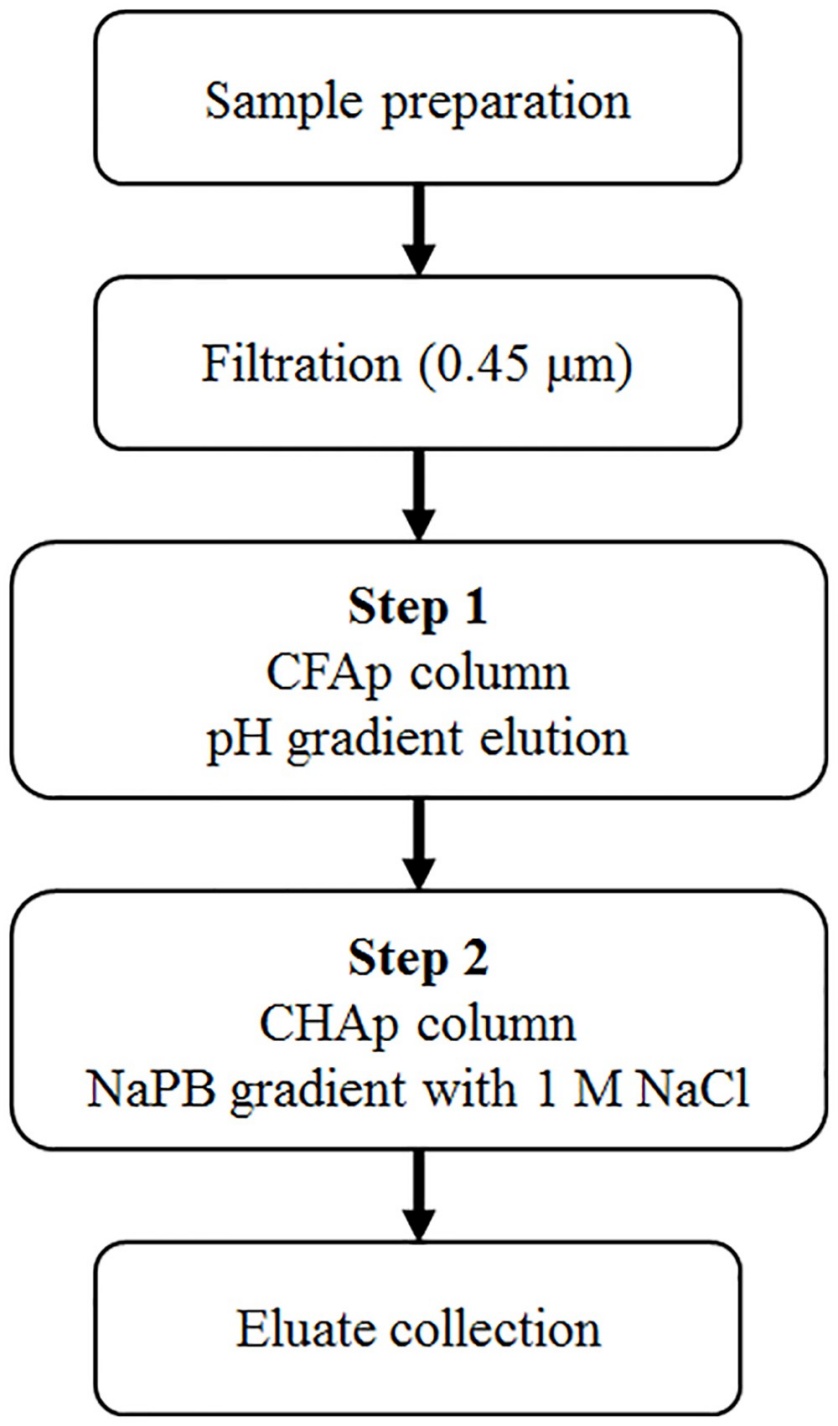
Standardized two-step purification procedure. CFAp, ceramic fluoroapatite; CHAp, ceramic hydroxyapatite; NaPB, sodium phosphate buffer.

We initially used one-step CHAp chromatography [16,17,29] to purify live attenuated Sabin type 2 virus. However, this proved unsuccessful because both protein and DNA contaminants could not be separated from the virus fraction using this method (data not shown). Therefore, we attempted to introduce separation by CFAp chromatography as the first step before conducting CHAp chromatography. The strategy of this two-step chromatographic procedure is outlined in Fig 1. In Step 1, virions were directly separated from the cell culture supernatant containing a complex protein mixture of infected cells by CFAp chromatography using pH gradient elution. In Step 2, the resulting virus fraction was separated by CHAp chromatography using NaPB gradient elution with a high concentration of sodium chloride (NaCl) to remove contaminated dsDNA from the fraction. We optimized each step as described below to separate the virus.

### Step 1: pH gradient elution of Sabin type 2 virus from a CFAp column

We separated three viruses [poliovirus Sabin type 2; dengue virus type 1 (Hawaii) (Department of Virology, Institute of Tropical Medicine, Nagasaki University, Japan); and influenza virus NYMC X-181 (National Institute for Biological Standards and Control, Hertfordshire, UK)] on a CHAp column by elution with a similar gradient of NaPB at different pH values (S1–S3 Figs). We found that the retention volumes of these viruses increased as the pH of the running buffer was lowered, regardless of the physicochemical properties of the virus; however, at pH 6.4, the Sabin type 2 virus fraction still contained protein contaminants (S1 Fig), whereas the dengue virus fraction appeared to have been separated from the protein fraction (S2 Fig). Therefore, we then attempted to apply CFAp chromatography, which shows similar separation to CHAp but allows a further reduction in the pH of the running buffer due to its greater tolerance to the acidic mobile phase (applicable pH range, 5–14) compared with CHAp (applicable pH range, 6.5–14) [25,26].

**Fig 2:**
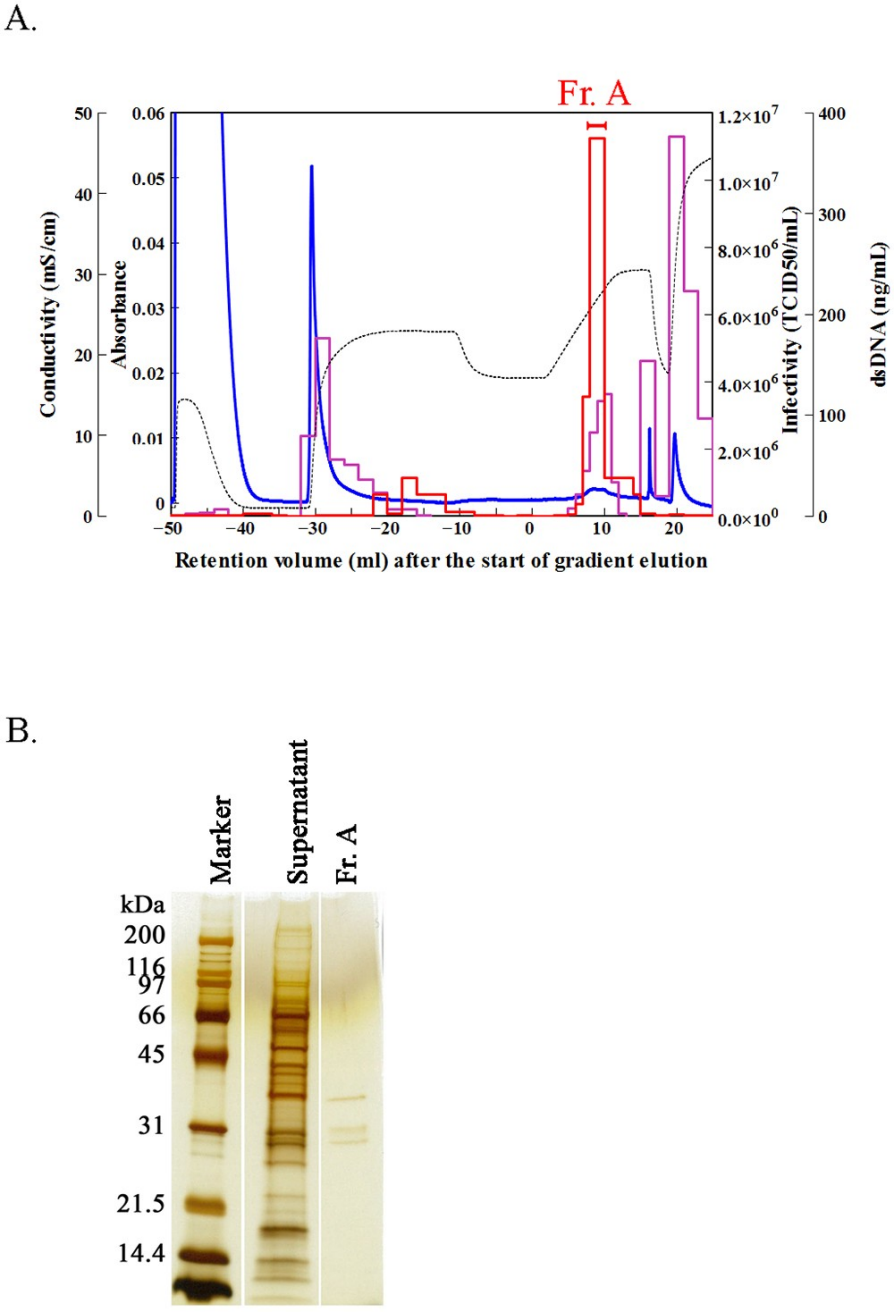
Purification of Sabin type 2 virus by pH gradient elution on a ceramic fluoroapatite (CFAp) column. (A) Chromatogram obtained under the following conditions: column, CFAp; sample (volume), cell culture supernatant containing Sabin type 2 virus (5 mL); column wash, 10 mM sodium phosphate buffer (NaPB; pH 6.4, 10 mL) and 300 mM NaPB (pH 6.4, 20 mL); equilibration, 300 mM NaPB (pH 5, 15 mL); elution, linear gradient at 300 mM NaPB from pH 5 to pH 8.2 for 10 mL; wash after separation, 300 mM NaPB (pH 8.2, 5 mL) and 600 mM NaPB (pH 8.2, 10 mL). Blue line, ultraviolet (UV) absorbance at 280 nm; black broken line, conductivity; red line, infectivity in median tissue culture infectious dose (TCID_50_); and purple line, double-stranded DNA (dsDNA) contents. Fr. A was pooled for the evaluation. (B) Sodium dodecyl sulfate–polyacrylamide gel electrophoresis (SDS–PAGE) analysis. The cell culture supernatant and pooled Fr. A were concentrated 10-fold and 30-fold, respectively, by ultrafiltration using a molecular weight cutoff of 10,000. The molecular weights of the marker proteins are given in kDa.

We separated Sabin type 2 virus from the cell culture supernatant on a CFAp column using a linear pH gradient from pH 5.0 to 8.2 in 300 mM NaPB because use of a pH gradient reduces the contact time between the low pH and the virus (Fig 2A). The mean retention volume of the virus was found to be 9.6 ± 0.44 mL (11 independent measurements, mean ± standard deviation), indicating that the separation was highly reproducible. The resulting fraction (Fr. A indicated in Fig 2A) showed a mean recovery of 94.1% ± 42.2% (11 independent measurements) in TCID_50_ and a mean protein removal rate of 91.87% (two independent measurements). Analysis of the fraction using SDS–PAGE showed that there were three bands at approximately 37, 32, and 30 kDa (Fig 2B), which agreed with the previously reported separation patterns of the capsid proteins VP1, VP2, and VP3 of Sabin type 2 virus [30], indicating that the virus had been separated from the protein contaminants. Nevertheless, the fraction still contained dsDNA (mean removal rate, 88.15%; two independent measurements) derived from the host cells that needed to be removed.

### Step 2: Separation of Sabin type 2 virus from dsDNAs by CHAp: Effect of NaCl addition to the mobile phase

We next examined whether a high concentration of NaCl in the mobile phase of Step 2 would allow dsDNA to be removed from the virus fraction based on the knowledge that excess sodium ions on the phosphate site of hydroxyapatite decrease the electrostatic repulsion between the phosphate site and DNA, causing an increase in the relative affinity of the calcium site for DNA [31]. To evaluate this, we separated two viruses (influenza virus NYMC X-181 and feline calicivirus) in the presence of 0–1.5 M NaCl. We found that the retention volume of dsDNA increased with increasing NaCl concentrations in the running buffer, whereas the retention volume of the virus particles decreased (S4 and S5 Figs). Thus, a high concentration of NaCl in the mobile phase proved effective in removing dsDNA from the virus fraction.

**Fig 3:**
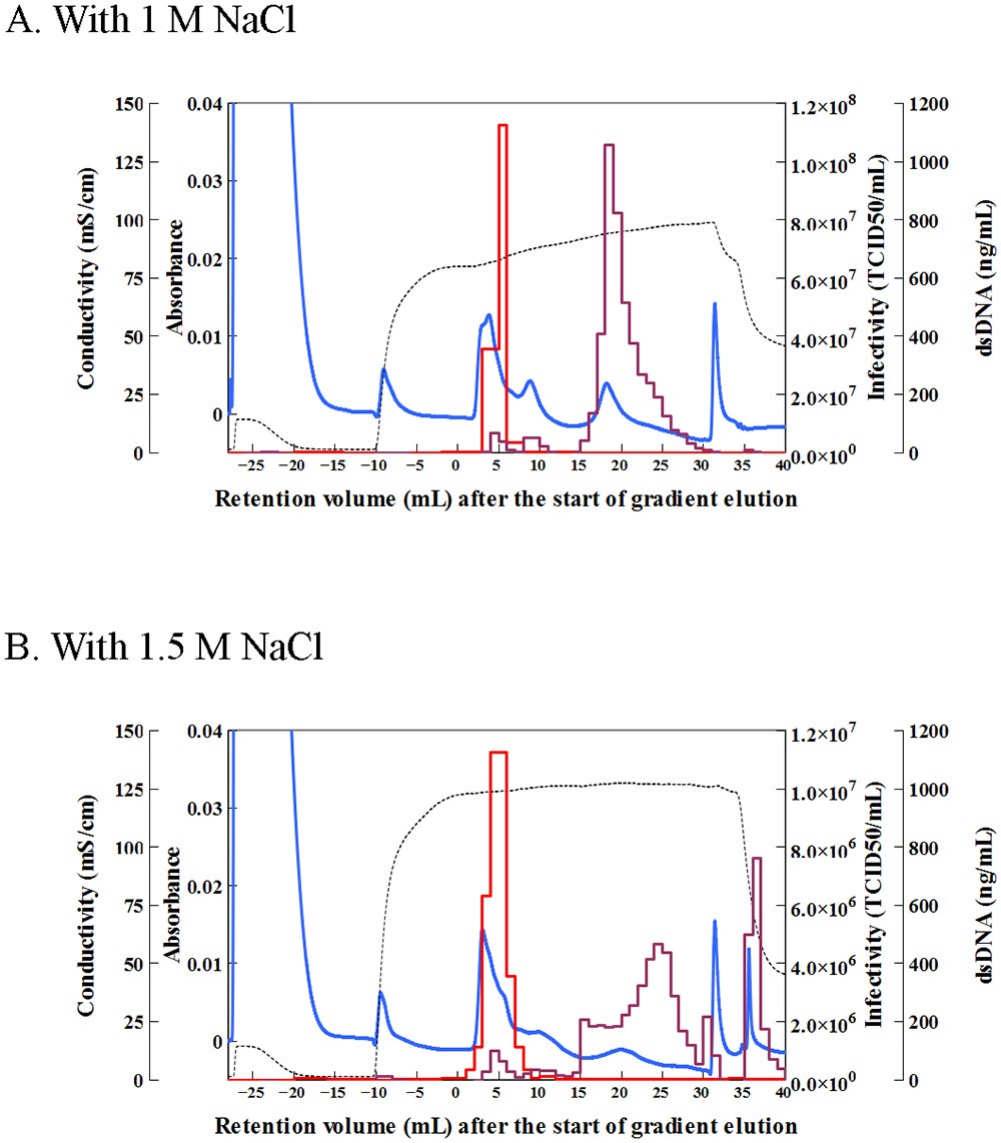
Chromatograms of Sabin type 2 virus-containing cell culture supernatant in the presence of NaCl. Separation was carried out in the presence of (A) 1 M NaCl and (B) 1.5 M NaCl under the following conditions: column, ceramic hydroxyapatite (CHAp); sample (volume), cell culture supernatant containing Sabin type 2 virus (5 mL); buffer pH, 7.2; column wash, 10 mM sodium phosphate buffer (NaPB; 9 mL); equilibration, 10 mM NaPB with NaCl (14 mL); elution, linear gradient from 10 mM to 600 mM NaPB with NaCl for 30 mL; wash after separation, 600 mM NaPB (10 mL). Lines are the same as in Fig 2. TCID_50_, median tissue culture infectious dose.

We used 1 and 1.5 M NaCl in the mobile phase to separate Sabin type 2 virus from dsDNA in the cell culture supernatant (Fig 3). An increased retention of dsDNA compared with the virus particle was obtained in both separations, indicating that the addition of 1 M NaCl was sufficient to remove dsDNA from the virus peak (removal rate, 97.32%). Furthermore, this separation was highly reproducible, with a virus retention volume after the start of gradient elution of 5.0 ± 0.5 mL (three independent measurements).

### Sequential two-step chromatographic purification of Sabin type 2 virus

**Fig 4:**
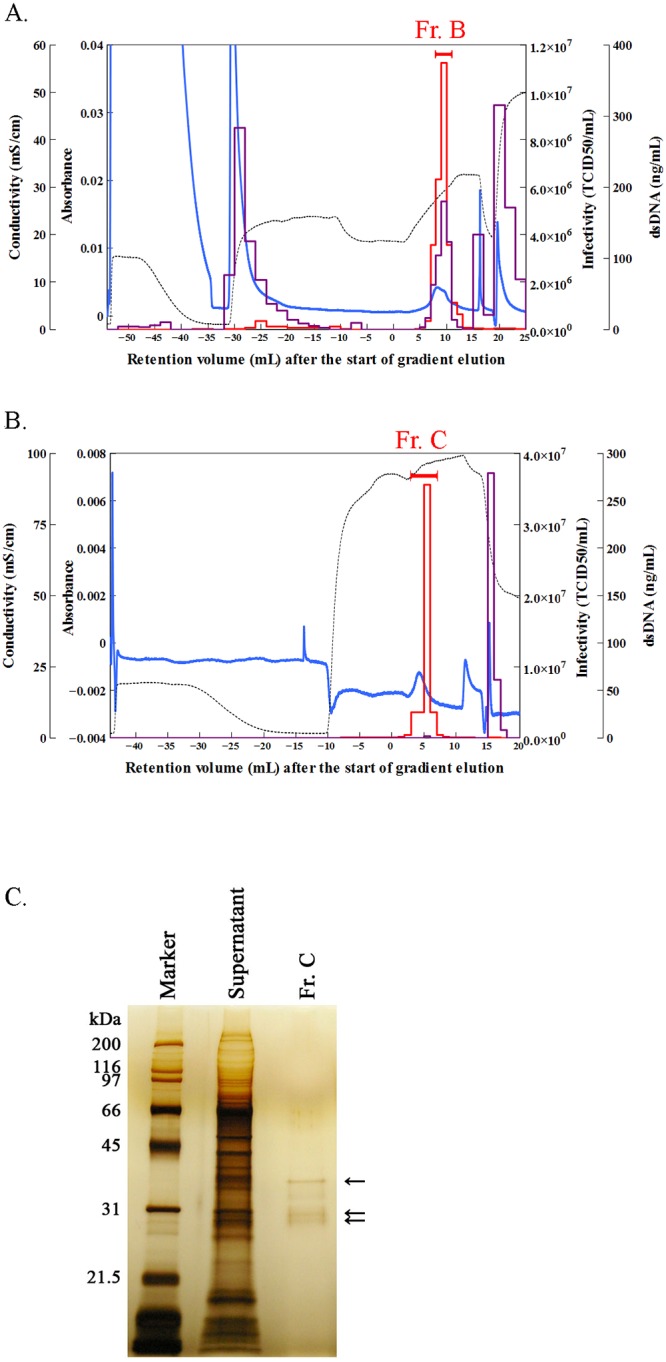
Sequential two-step purification of Sabin type 2 virus. (A) Purification of Sabin type 2 virus by pH gradient elution on a ceramic fluoroapatite (CFAp) column (Step 1). Column, CFAp; sample (volume), cell culture supernatant containing Sabin type 2 virus (10 mL); wash, 10 mM sodium phosphate buffer (NaPB) (pH 6.4, 9 mL) and 300 mM NaPB (pH 6.4, 20 mL); equilibration, 15 mL of 300 mM NaPB (pH 5); elution, linear gradient from pH 5 to pH 8.2 at 300 mM NaPB for 10 mL; wash after separation, 300 mM NaPB (pH 8.2, 5 mL) and 600 mM NaPB (pH 8.2, 10 mL). Lines are the same as in Fig 2. Fr. B was pooled and further purified in Step 2. (B) Removal of double-stranded DNA (dsDNA) from the Fr. B fraction by NaPB elution with 1 M NaCl on a ceramic hydroxyapatite (CHAp) column (Step 2). Column, CHAp; sample (volume), Fr. B obtained in (A) diluted 6.7-fold with 0.9% NaCl (17 mL); buffer, pH 7.2; column wash, 10 mM NaPB (13 mL); equilibration, 10 mM NaPB (14 mL) with 1 M NaCl; elution, linear gradient from 10 mM to 187 mM NaPB with 1 M NaCl for 10 mL; wash, 600 mM NaPB (10 mL). Fr. C was pooled for further evaluation. (C) Sodium dodecyl sulfate–polyacrylamide gel electrophoresis (SDS–PAGE) analysis of the pooled fraction obtained through the two-step purification process. The pooled Fr. C fraction obtained in (B) and the cell culture supernatant were concentrated 100- and 10-fold, respectively, by ultrafiltration using a molecular weight cutoff of 10,000. The sizes are given in kDa on the left. Arrows indicate the major proteins in Fr. C. TCID_50_, median tissue culture infectious dose.

We performed the two-step sequential procedure (Steps 1 and 2 described above) to isolate Sabin type 2 virus from the cell culture supernatant. A representative case is shown in Fig 4 and S1 Table. The virus particles were separated by pH gradient elution on a CFAp column (Fig 4A), and the resulting viral fraction “Fr. B” was pooled, relying on the average retention volume that was observed above. Fr. B was then diluted 6.7-fold with 0.9% NaCl, loaded onto a CHAp column, and eluted with a linear gradient of NaPB (pH 7.2) with 1 M NaCl (Fig 4B). The peak TCID_50_ was detected at 3–7 mL (Fr. C) of the retention volume and contained highly purified capsid proteins (Fig 4B and 4C).

## Discussion

**Fig 5:**
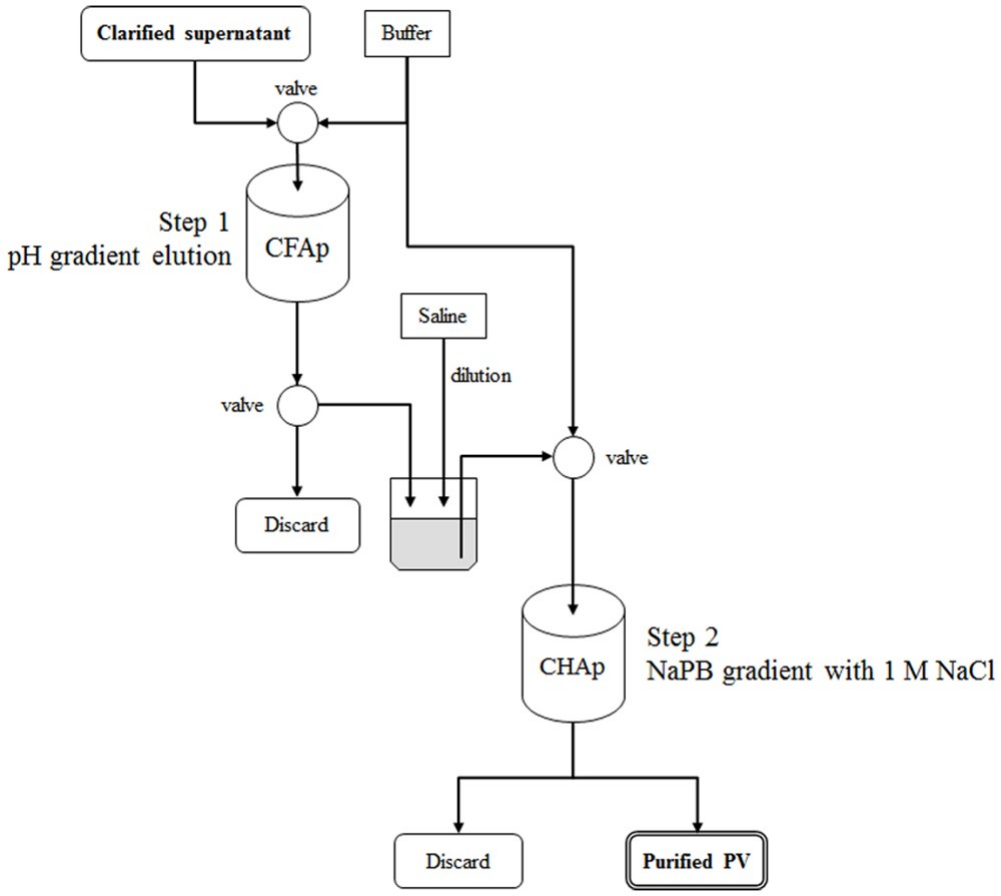
Tandem column system. CFAp, ceramic fluoroapatite; CHAp, ceramic hydroxyapatite; NaPB, sodium phosphate buffer; PV, poliovirus.

When manufacturing vaccines for use in clinical trials, preclinical studies, or commercial medicines, the aim is to minimize impurities and maximize the recovery of the vaccine during purification. The impurities are typically DNA and proteins derived from host cells, components of the cell culture medium, and/or some ligands released from purification process [32]. In this study, we successfully purified Sabin type 2 virus directly from the cell culture supernatant without using any concentrating or buffer exchange processes, achieving the mean recovery rate in TCID_50_ of 58.7% ± 30.0%, and the mean removal rates of proteins and dsDNA of 99.95 ± 0.006% and 99.99% ± 0.003%, respectively, (three independent experiments). The current permissible host cell protein and DNA levels in viral vaccines as recommended by the World Health Organization (WHO) are <10 μg and <10 ng per 40 D-antigen units, respectively [33]. Because our analysis was not based on the measurement of D-antigen unit-based purity, we were unable to compare between purities obtained in this study and those recommended by WHO standards. This limitation needs to be addressed when considering future scale-ups and automation. In Step 1, the cell culture supernatant was loaded directly onto a CFAp column and eluted with a pH gradient. In Step 2, the resulting virus fraction was diluted with 0.9% NaCl, loaded on a CHAp column, and eluted with an NaPB gradient in the presence of a high concentration of NaCl. Because both Steps 1 and 2 were highly reproducible, the viral fraction could be obtained without the need for any detection processes. This retention-volume-dependent fractionation should reduce the time and cost required to make vaccines. Furthermore, although the procedure currently contains multistep and offline purification processes, it should be possible to automate all the purification steps in the future. This two-step chromatographic purification is also easier to scale up than other current procedures [34] because it was constructed using fully scalable processes (Fig 5).

Two-step chromatographic purification may also be useful for purifying other poliovirus strains, such as Salk and Sabin types 1 and 3, as well as other nonenveloped viruses, including hepatitis A virus, norovirus, and feline calicivirus. However, this process does have some methodological limitations because Step 1 uses a low pH that will disrupt some viruses. Consequently, it may not be applicable for the purification of flaviviruses, which are unstable at acidic pH values [35].

In addition to their role in vaccine production, infective virus purification techniques also play an important role in the field of gene therapy. In particular, there is an urgent clinical need for the large-scale purification of adenovirus because this virus is considered one of the most suitable platforms for long-term gene transduction [36]. Several other gene therapies have also yielded promising results for the use of virus vectors that express clinically relevant genes [37]. For example, oncolytic adenovirus has been drawing attention recently as an antitumor medicine [38], and dozens of studies using this virus are now registered in ClinicalTrials.Gov (https://clinicaltrials.gov/), a database of clinical studies conducted around the world [39]. This new purification method should be appropriate for adenoviruses that are resistant to acidic purification conditions.

## Supporting information

S1 Fig Chromatograms of Sabin type 2 virus-containing cell culture supernatant separated on a ceramic hydroxyapatite (CHAp) column at different pH values. Separation occurred at buffer pH values of (A) 6.4, (B) 7.2, and (C) 8.2. Column, CHAp (40 μm); sample (volume), cell culture supernatant containing Sabin type 2 virus (5 mL); column wash and equilibration, 10 mM sodium phosphate buffer (NaPB; 11 mL); elution, linear gradient from 10 mM to 300 mM NaPB for 20 mL; wash after separation, 600 mM NaPB (8 mL). Blue line, ultraviolet (UV) absorbance at 280 nm; black broken line, conductivity; red line, infectivity in median tissue culture infectious dose (TCID_50_); and purple line, double-stranded DNA (dsDNA) contents.
(TIF)

S2 Fig Chromatograms of dengue virus type 1-containing cell culture supernatant separated on a ceramic hydroxyapatite (CHAp) column at different pH values. Separation occurred at buffer pH values of (A) 6.4, (B) 7.2, and (C) 8.2. Column, CHAp (40 μm); sample (volume), cell culture supernatant containing dengue virus type 1 (10 mL); column wash and equilibration, 10 mM sodium phosphate buffer (NaPB; 10 mL); elution, linear gradient from 10 mM to 300 mM NaPB for 30 mL and from 300 mM to 600 mM for 8 mL; wash after separation, 600 mM NaPB (5 mL). Lines are the same as in S1 Fig except for the red line, which indicates virus activity in the hemagglutination (HA) assay.
(TIF)

S3 Fig Chromatograms of influenza virus NYMC X-181 containing cell culture supernatant separated on a ceramic hydroxyapatite (CHAp) column at different pH values. Separation occurred at buffer pH values of (A) 6.5, (B) 6.8, and (C) 7.5. Column, CHAp (40 μm); sample (volume), cell culture supernatant containing influenza virus NYMC X-181 (5 mL); column wash and equilibration, 10 mM sodium phosphate buffer (NaPB; 15 mL); elution, linear gradient from 10 mM to 600 mM NaPB for 30 mL; wash, 600 mM NaPB (5 mL). Lines are the same as in S1 Fig except for the red line, which indicates virus activity in the hemagglutination (HA) assay.
(TIF)

S4 Fig Chromatograms of influenza virus NYMC X-181 containing cell culture supernatant in the presence of NaCl. The buffer contained (A) 0 M, (B) 0.14 M, (C) 0.5 M, and (D) 1 M NaCl. Column, ceramic hydroxyapatite (CHAp); sample (volume), cell culture supernatant containing influenza virus NYMC X-181 (5 mL); buffer pH, 7.5; column wash and equilibration, 5 mM sodium phosphate buffer (NaPB) with NaCl (15 mL); elution, linear gradient from 5 mM to 600 mM NaPB with NaCl for 30 mL; wash after separation, 600 mM NaPB (5 mL). Lines are the same as in S1 Fig except for the red line, which indicates virus activity in the hemagglutination (HA) assay.
(TIF)

S5 Fig Chromatograms of feline calicivirus-containing cell culture supernatant in the presence of NaCl. The buffer contained (A) 0 M, (B) 1 M, and (C) 1.5 M NaCl. Column, ceramic hydroxyapatite (CHAp); sample (volume), cell culture supernatant containing feline calicivirus A391 (1 mL); buffer pH, 7.2. (A) Equilibration, 20 mL of 200 mM sodium phosphate buffer; elution, linear gradient from 200 mM to 600 mM NaPB for 25 mL. (B and C) Equilibration, 10 mM NaPB with NaCl; elution, linear gradient from 10 mM to 600 mM NaPB with NaCl for 20 mL. Before and after separation, the column was washed with 10 mM NaPB (19 mL) and 600 mM NaPB (10 mL), respectively, with NaCl at the concentrations indicated. Lines are the same as in S1 Fig. TCID_50_, median tissue culture infectious dose.
(TIF)

S1 Table Evaluation of the obtained fractions. TCID_50_, median tissue culture infectious dose; dsDNA, double-stranded DNA.
(DOCX)

S1 Appendix (DOCX)
