# Supplementary figures and images for: Sequential two-step chromatographic purification of infectious poliovirus using ceramic fluoroapatite and ceramic hydroxyapatite columns
Source: PLoS One. 2019 Sep 19;14(9):e0222199. doi: 10.1371/journal.pone.0222199 (PMC6752803; doi:10.1371/journal.pone.0222199)

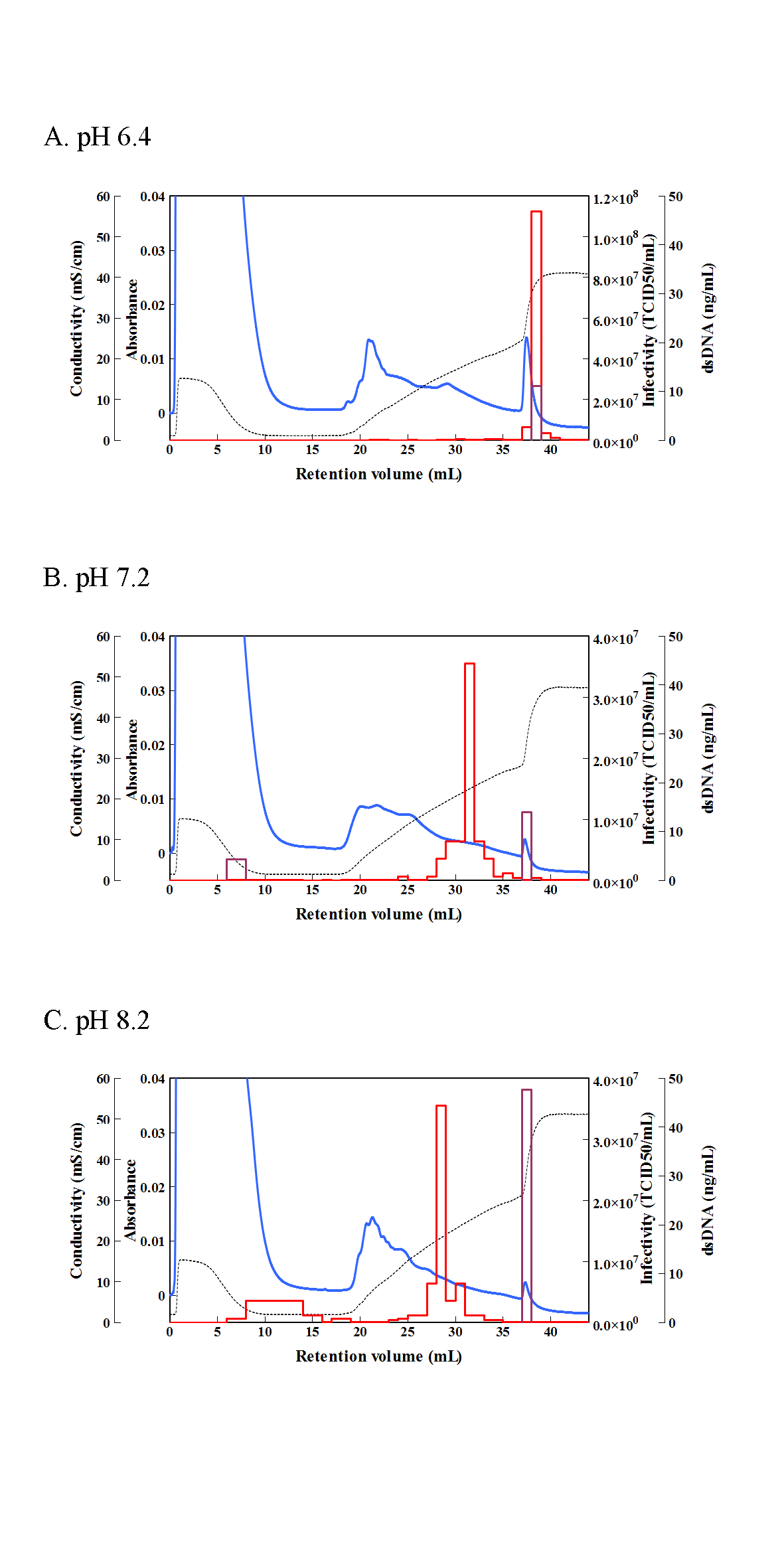

Supplement: S1 Fig — Separation occurred at buffer pH values of (A) 6.4, (B) 7.2, and (C) 8.2. Column, CHAp (40 μm); sample (volume), cell culture supernatant containing Sabin type 2 virus (5 mL); column wash and equilibration, 10 mM sodium phosphate buffer (NaPB; 11 mL); elution, linear gradient from 10 mM to 300 mM NaPB for 20 mL; wash after separation, 600 mM NaPB (8 mL). Blue line, ultraviolet (UV) absorbance at 280 nm; black broken line, conductivity; red line, infectivity in median tissue culture infectious dose (TCID50); and purple line, double-stranded DNA (dsDNA) contents. (TIF) [file pone.0222199.s001.tif]

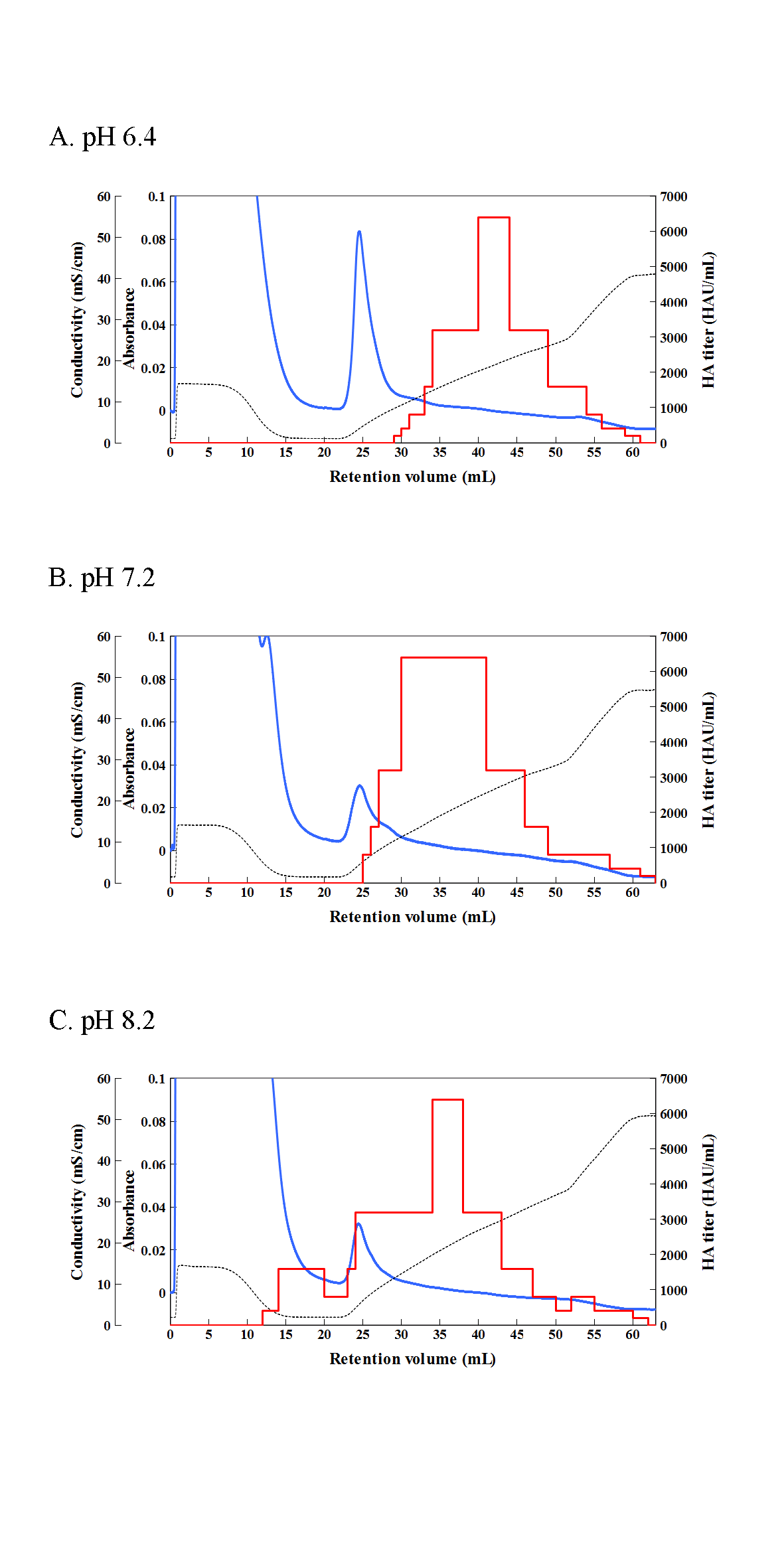

Supplement: S2 Fig — Separation occurred at buffer pH values of (A) 6.4, (B) 7.2, and (C) 8.2. Column, CHAp (40 μm); sample (volume), cell culture supernatant containing dengue virus type 1 (10 mL); column wash and equilibration, 10 mM sodium phosphate buffer (NaPB; 10 mL); elution, linear gradient from 10 mM to 300 mM NaPB for 30 mL and from 300 mM to 600 mM for 8 mL; wash after separation, 600 mM NaPB (5 mL). Lines are the same as in S1 Fig except for the red line, which indicates virus activity in the hemagglutination (HA) assay. (TIF) [file pone.0222199.s002.tif]

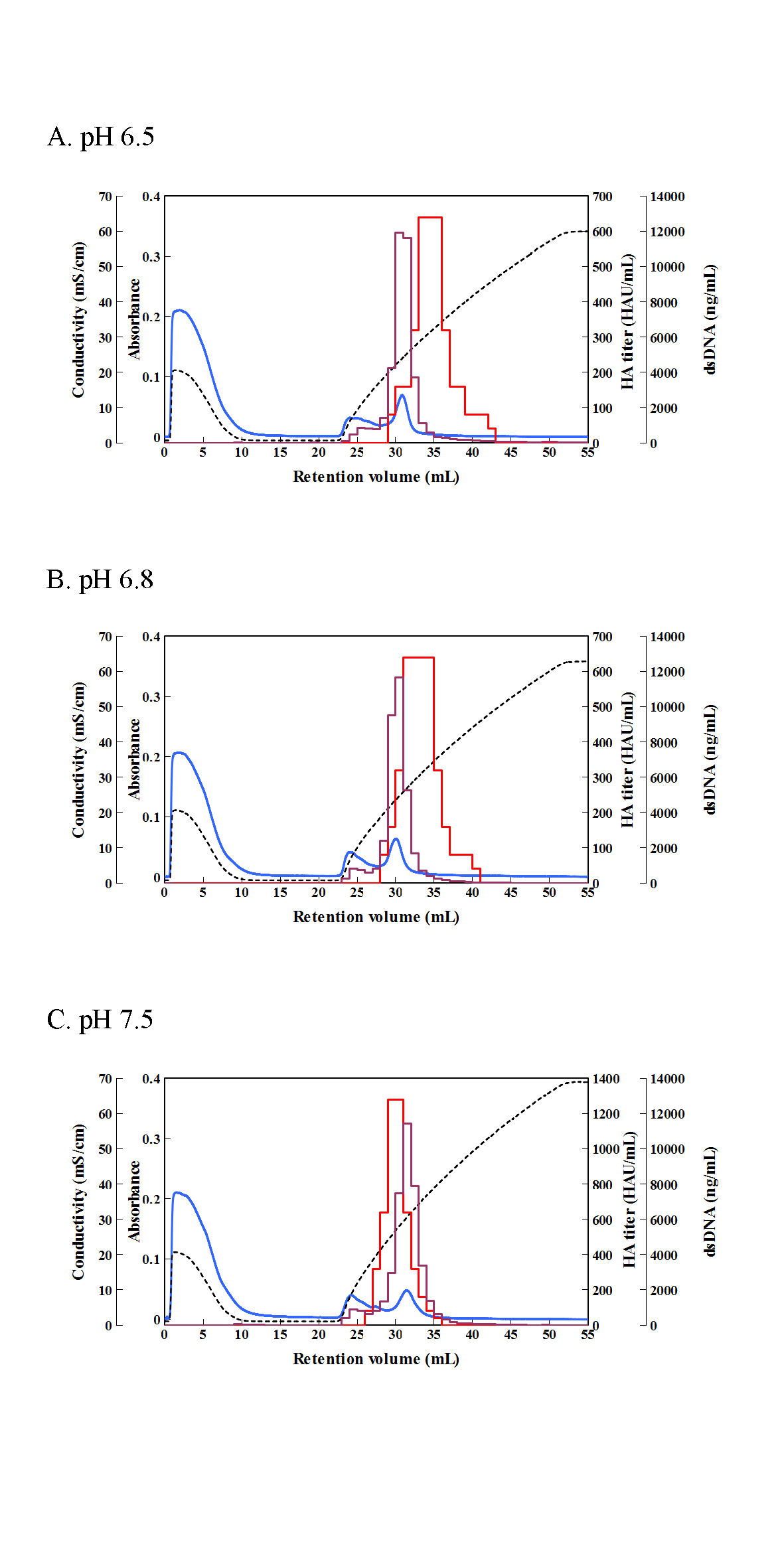

Supplement: S3 Fig — Separation occurred at buffer pH values of (A) 6.5, (B) 6.8, and (C) 7.5. Column, CHAp (40 μm); sample (volume), cell culture supernatant containing influenza virus NYMC X-181 (5 mL); column wash and equilibration, 10 mM sodium phosphate buffer (NaPB; 15 mL); elution, linear gradient from 10 mM to 600 mM NaPB for 30 mL; wash, 600 mM NaPB (5 mL). Lines are the same as in S1 Fig except for the red line, which indicates virus activity in the hemagglutination (HA) assay. (TIF) [file pone.0222199.s003.tif]

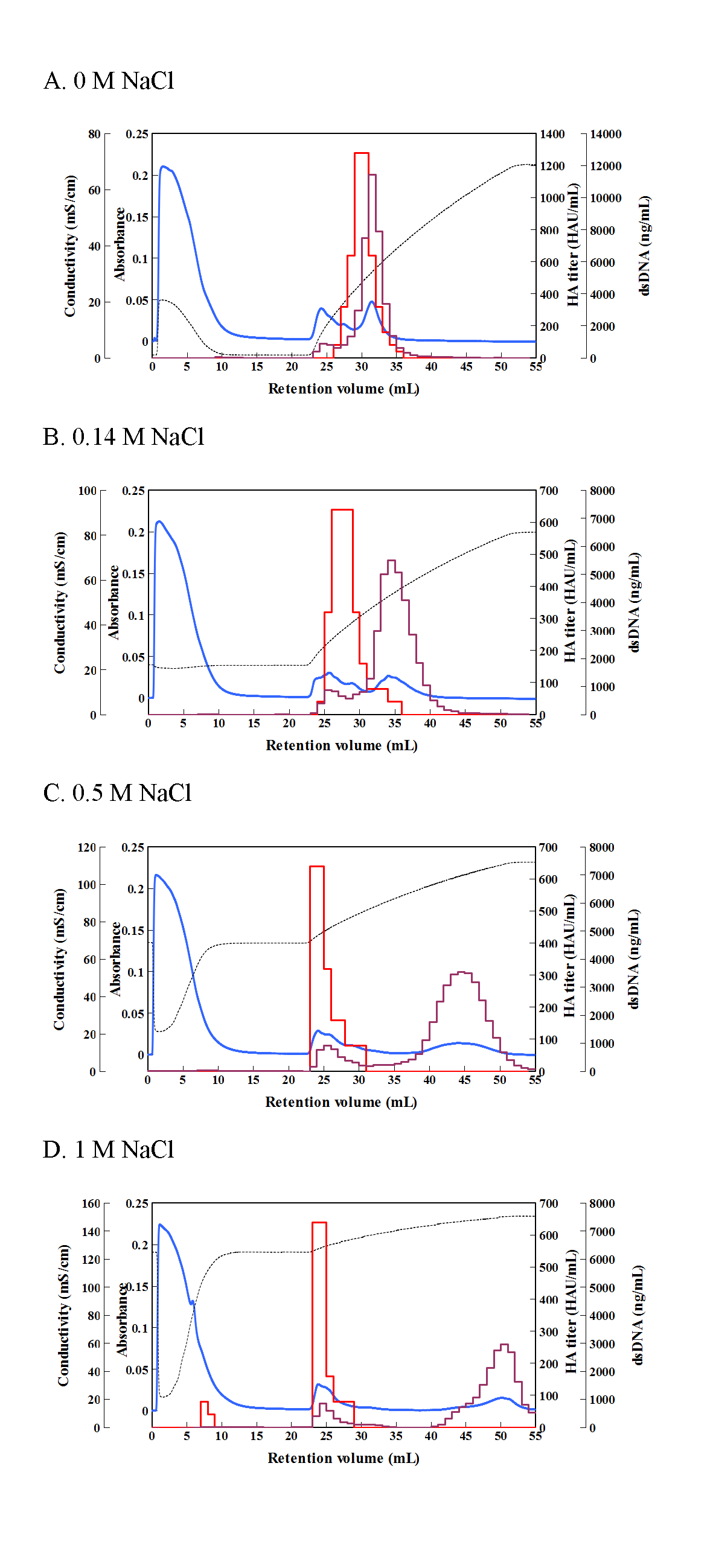

Supplement: S4 Fig — The buffer contained (A) 0 M, (B) 0.14 M, (C) 0.5 M, and (D) 1 M NaCl. Column, ceramic hydroxyapatite (CHAp); sample (volume), cell culture supernatant containing influenza virus NYMC X-181 (5 mL); buffer pH, 7.5; column wash and equilibration, 5 mM sodium phosphate buffer (NaPB) with NaCl (15 mL); elution, linear gradient from 5 mM to 600 mM NaPB with NaCl for 30 mL; wash after separation, 600 mM NaPB (5 mL). Lines are the same as in S1 Fig except for the red line, which indicates virus activity in the hemagglutination (HA) assay. (TIF) [file pone.0222199.s004.tif]

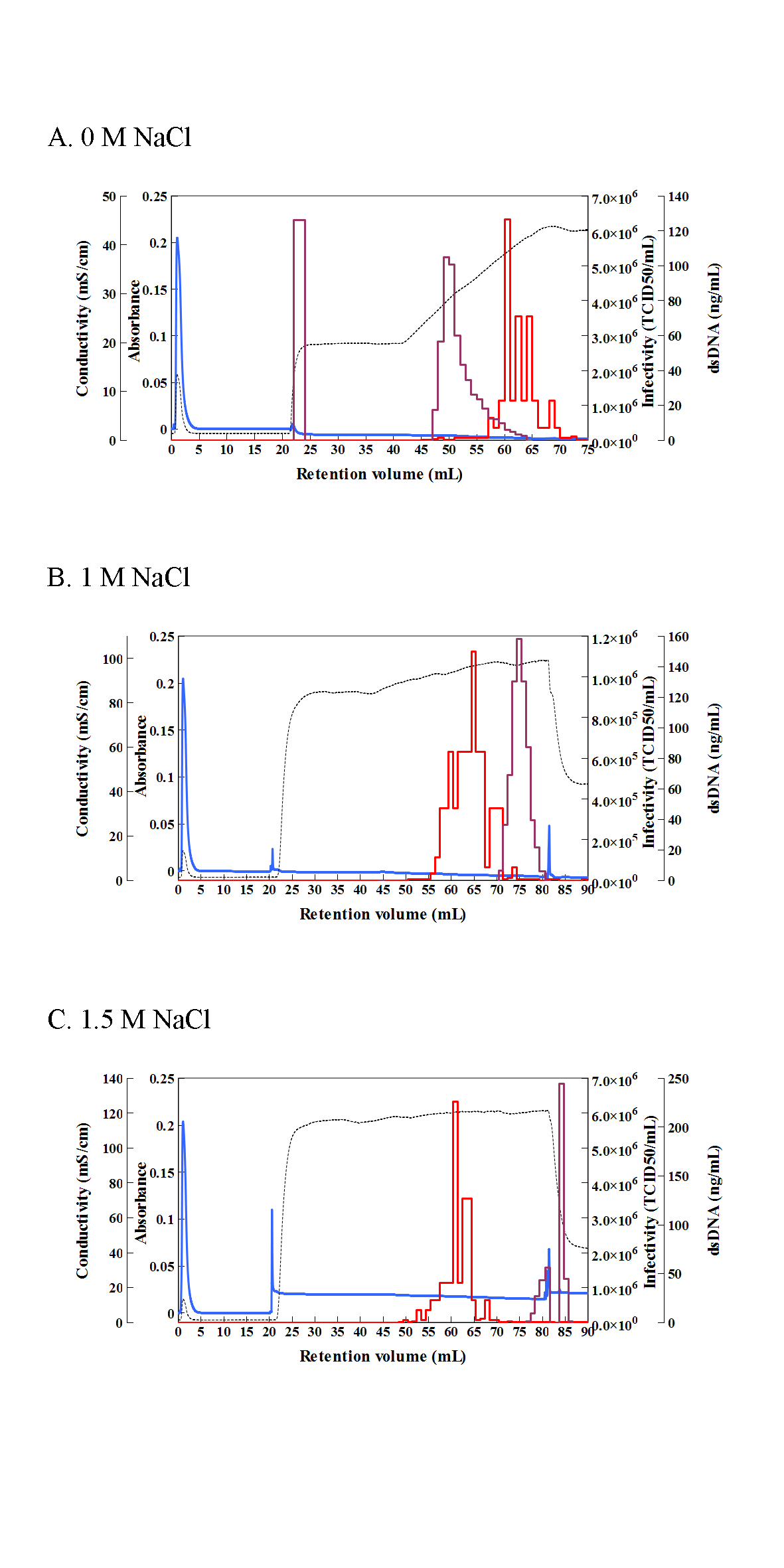

Supplement: S5 Fig — The buffer contained (A) 0 M, (B) 1 M, and (C) 1.5 M NaCl. Column, ceramic hydroxyapatite (CHAp); sample (volume), cell culture supernatant containing feline calicivirus A391 (1 mL); buffer pH, 7.2. (A) Equilibration, 20 mL of 200 mM sodium phosphate buffer; elution, linear gradient from 200 mM to 600 mM NaPB for 25 mL. (B and C) Equilibration, 10 mM NaPB with NaCl; elution, linear gradient from 10 mM to 600 mM NaPB with NaCl for 20 mL. Before and after separation, the column was washed with 10 mM NaPB (19 mL) and 600 mM NaPB (10 mL), respectively, with NaCl at the concentrations indicated. Lines are the same as in S1 Fig. TCID50, median tissue culture infectious dose. (TIF) [file pone.0222199.s005.tif]
