# Supplementary material for: Sequential two-step chromatographic purification of infectious poliovirus using ceramic fluoroapatite and ceramic hydroxyapatite columns
Source: PLoS One. 2019 Sep 19;14(9):e0222199. doi: 10.1371/journal.pone.0222199 (PMC6752803; doi:10.1371/journal.pone.0222199)
Supplement: S1 Table — TCID50, median tissue culture infectious dose; dsDNA, double-stranded DNA. (DOCX) [file pone.0222199.s006.docx]

**S1 Table. Evaluation of the obtained fractions.**

|  | **Infectivity** | | **Protein** | | | **dsDNA** | | |
| --- | --- | --- | --- | --- | --- | --- | --- | --- |
|  | **Total TCID_50_** | **Recovery (%)** | **Conc.**  **(μg/mL)** | **Total protein (μg)** | **Removal**  **(%)** | **Conc.**  **(ng/mL)** | **Total DNA (ng)** | **Removal**  **(%)** |
| **Supernatant**  **(load)** | 6.3×10^7^ | 100 | 728 | 7,277 | - | 443 | 4,425 | - |
| **Step 1**  **Fr. B** | 2.1×10^7^ | 33 | 2.9 | 8.8 | 99.88 | 195 | 584 | 86.79 |
| **Step 2**  **Fr. C** | 4.6×10^7^ | 73 | 1.0 | 3.9 | 99.95 | <0.098 | <0.391 | >99.99 |
