# Supplementary material for: Sequential two-step chromatographic purification of infectious poliovirus using ceramic fluoroapatite and ceramic hydroxyapatite columns
Source: PLoS One. 2019 Sep 19;14(9):e0222199. doi: 10.1371/journal.pone.0222199 (PMC6752803; doi:10.1371/journal.pone.0222199)
Supplement: S1 Appendix — (DOCX) [file pone.0222199.s007.docx]

**S1 Appendix**

**Materials and Methods**

**Preparation of the Dengue virus**

Dengue virus type 1 and C6/36 cells were provided by Professor K. Morita of Department of Virology, Institute of Tropical Medicine, Nagasaki University, Japan. C6/36 cells were cultured in 225-cm^2^ flasks (Sumitomo Bakelite Co., Ltd., Tokyo, Japan) pre-coated with poly-L-Lysine (Sigma-Aldrich Co., St. Louis, MO, USA) in modified Eagle Minimum Essential Medium (modified EMEM; MP Biomedicals, Irvine, CA, USA) containing 10% fetal bovine serum (FBS; Thermo Fisher Scientific Inc.) at 28°C for 1 week [1]. Dengue virus type 1, strain Hawaii was inoculated onto the cell monolayer in 75 mL of modified EMEM containing 0.5% FBS and MEM vitamin solution (Thermo Fisher Scientific Inc., Waltham, MA, USA) and was cultured at 28°C. The culture supernatant was collected and filtered via a 0.45-μm filter to remove cells and cell debris.

**Hemagglutination (HA) test for dengue virus**

Goose red blood cells (GRBCs; Nippon BIotest Laboratories Inc., Saitama, Japan) were washed thrice with saline [1]. GRBCs were suspended in the virus after adjusting a diluent containing 150 mM NaCl and 200 mM sodium phosphate at pH 6.2 to a final concentration of 0.33%. Each fraction was diluted 10-fold, and 50 μL of the diluted sample was further serially diluted 2-fold with 0.4% bovine serum albumin (Wako Pure Chemical Industries, Ltd., Osaka, Japan) in BS9 (120 mM NaCl, 50 mM boric acid, and 24 mM sodium hydrate) in U-bottom 96-well plates (Corning Inc., NY, USA). Thereafter, 50 μL of the 0.33% GRBC suspension was added into each well, the plates were gently mixed, incubated at 37°C for 30 min. The HA titer was the highest dilution of the virus that showed an agglutination pattern on the bottom of the well.

**Preparation of the influenza virus**

Influenza virus NYMC X-181 (National Institute for Biological Standards and Control, Hertfordshire, UK) was inoculated onto MDCK (The American Type Culture Collection, Manassas, VA, USA) cell monolayer in 225-cm^2^ flasks (Sumitomo Bakelite Co., Ltd.) in 75 mL DMEM/F-12 medium (Thermo Fisher Scientific Inc.) supplemented with 5% CO_2_ at 37°C. The cell culture supernatant was collected and passed via a 0.45-μm filter using vacuum to remove cells and cell debris.

**Hemagglutination (HA) test for influenza virus**

Chicken red blood cells (CRBCs; Nippon BIotest Laboratories Inc.) were washed thrice with phosphate buffered saline (PBS). CRBCs were suspended in PBS at a final concentration of 0.5%. Each fraction was diluted 10-fold, and 50 μL of the diluted samples were further serially diluted 2-fold with PBS in U-bottom 96-well plates (Corning Inc.). Thereafter, 50 μL of 0.5% CRBC suspension was added into each well, the plates were gently mixed, and incubated at 37°C for 30 min. The HA titer was the highest dilution of virus that showed the agglutination pattern on the bottom of the well.

**Preparation of the feline calicivirus**

Feline calicivirus, strain A391 and Cat S+L− cell were kindly supplied by Dr. N. Hirano of Iwate University, Japan [2]. Feline calicivirus was inoculated onto Cat S+L− cell monolayers in 225-cm^2^ flasks (Sumitomo Bakelite Co., Ltd.) with 75 mL of minimum essential medium (MEM; Thermo Fisher Scientific Inc.) containing L-glutamine (2 mM; Thermo Fisher Scientific Inc.) supplemented with 5% CO_2_ at 37°C. The cell culture supernatant was collected and passed via a 0.45-μm filter using vacuum to remove cells and cell debris.

**Median tissue culture infectious dose (TCID50) assay for feline calicivirus**

The feline calicivirus titer was determined using TCID50. Cat S+L− cells (100 μL, 1.5 × 10^5^ cells/mL) were cultured in 96-well microplates with MEM containing 10% FBS at 37°C for 1 day. Each fraction was 10-fold serially diluted using MEM containing 10% FBS, and the resulting diluents (50 μL) were inoculated into each well (*n* = 3) and cultured further for 1 week. Each microplate well was examined under a light microscope for the occurrence of cytopathic effects. Titers were calculated using the Reed–Muench method [3].

**References**

1. Kurosawa Y, Saito M, Kobayashi S, Okuyama T. Purification of dengue virus particles by one-step ceramic hydroxyapatite chromatography. World J Vaccines. 2012;02: 155-160.
2. Hirano N, Sato R, Matsuda Y. A survey of feline respiratory infections. Nihon Juigaku Zasshi. 1986;48: 423-427.
3. Ballew HC. Neutralization. In: Specter S, Hodinka RL, Young SA, editors. 3^rd^ ed. Clinical virology manual. Washington DC: ASM Press; 2000. pp. 131.
